# Ferroptosis-related gene expression in the pathogenesis of preeclampsia

**DOI:** 10.3389/fgene.2022.927869

**Published:** 2022-08-17

**Authors:** Yuzhen Ding, Xiaofeng Yang, Xiaoxue Han, Meiting Shi, Lu Sun, Mengyuan Liu, Ping Zhang, Zhengrui Huang, Xiuli Yang, Ruiman Li

**Affiliations:** ^1^ Department of Obstetrics and Gynecology, The First Affiliated Hospital of Jinan University, Guangzhou, China; ^2^ Department of Obstetrics and Gynecology, The Sixth Affiliated Hospital of Jinan University, Dongguan, China

**Keywords:** preeclampsia, ferroptosis, placenta, bioinformatics, WGCNA

## Abstract

**Background:** Preeclampsia (PE) is one of the leading causes of maternal and fetal morbidity and mortality worldwide. Placental oxidative stress has been identified as a major pathway to the development of PE. Ferroptosis is a new form of regulated cell death that is associated with iron metabolism and oxidative stress, and likely mediates PE pathogenesis. The aim of the study was to identify the key molecules involved in ferroptosis to further explore the mechanism of ferroptosis in PE.

**Methods:** Gene expression data and clinical information were downloaded from the GEO database. The limma R package was used to screen differentially expressed genes (DEGs) and intersected with ferroptosis genes. The GO and KEGG pathways were then analyzed. Next, hub genes were identified *via* weighted gene co-expression network analysis (WGCNA). Receiver operating curves (ROCs) were performed for diagnostic and Pearson’s correlation of hub genes and clinicopathological characteristics. Immunohistochemistry and Western blot analysis were used to verify the expression of hub genes.

**Results:** A total of 3,142 DEGs were identified and 30 ferroptosis-related DEGs were obtained. In addition, ferroptosis-related pathways were enriched by GO and KEGG using DEGs. Two critical modules and six hub genes that were highly related to diagnosis of PE were identified through WGCNA. The analysis of the clinicopathological features showed that NQO1 and SRXN1 were closely correlated with PE characteristics and diagnosis. Finally, Western blot and immunohistochemistry analysis confirmed that the expression of the SRXN1 protein in the placental tissue of patients with PE was significantly elevated, while the expression of NQO1 was significantly decreased.

**Conclusions:** SRXN1 and NQO1 may be key ferroptosis-related proteins in the pathogenesis of PE. The study may provide a theoretical and experimental basis for revealing the pathogenesis of PE and improving the diagnosis of PE.

## Introduction

Preeclampsia (PE) is a pregnancy-related disorder and leading cause of maternal and fetal morbidity and mortality, affecting 3–8% of pregnancies worldwide (Guo et al., 2020). Epidemiological studies have shown that the occurrence and development of preeclampsia are related to the delay of female reproductive age, the increase in female obesity rate, and the popularization of assisted reproductive technology (Mccance et al., 2010; Almasi-Hashiani et al., 2019). The pathogenesis of preeclampsia is complex and involves many different mechanisms including immune imbalance, vascular endothelial cell damage, and inflammatory processes (Lawlor et al., 2012). However, it is well established that the reduced proliferation and abnormal invasion of trophoblasts lead to inadequate spiral artery remodeling and thereby to placenta ischemia and hypoxia (Staff et al., 2022). Numerous studies showed that programmed cell death plays an important role in placental physiology and trophoblast injury (Kasture et al., 2021). Ferroptosis, an iron-dependent form of programmed cell death, has been identified in preeclampsia, but the mechanism of ferroptosis in the placenta remains unclear (Ng et al., 2019).

Ferroptosis was first described in 2012 as a novel form of cell death, which was characterized by iron-dependent accretion of lipid peroxidation by reactive oxygen species (ROS) (Dixon et al., 2012). In recent years, ferroptosis has been shown to play a key role in metabolic diseases, cardiomyopathy, neurodegeneration, ischemia–reperfusion injury, and the effects of cancer immunotherapy (Fang et al., 2019; Khan et al., 2022). Therefore, targeting ferroptosis has become a hot research topic and can design new therapeutics and disease prevention measures. Recent studies have revealed that this process is related to ischemia/reperfusion during placental development (Beharier et al., 2020). Several studies have identified some key molecules that change after ferroptosis. Recent work indicated that ferritin genes are highly expressed in almost all cell types at the maternal–fetal interface, which indicates that intracellular iron storage and prevention of iron toxicity may be important at the maternal–fetal interface (Pavličev et al., 2017). Some studies found that trophoblast cells may be more prone to ferroptosis than other cell types at the maternal–fetal interface due to the high expression of *Lpcat3* and *Sat1* during placental development (Ou et al., 2016; Jiang et al., 2021; Lee et al., 2021). Recently, several studies have reported that *PLA2G6* is also highly expressed in human placental trophoblasts and can be used as a novel regulator of trophoblast ferroptosis (Beharier et al., 2020). Altogether, it remains to be fully elucidated how cellular ferroptosis is regulated and how these modifications involved in the development of preeclampsia. Currently, many ferroptosis-related genes have not yet been found, thus further studies of ferroptosis genes are urgently needed.

Currently, there are no bioinformatic studies on the mechanism of ferroptosis genes in the pathophysiology of preeclampsia. Therefore, we used data mining and data analysis approach to identify differentially expressed genes (DEGs) in placenta tissue between pregnant women with preeclampsia and healthy pregnant women. Weighted gene co-expression network analysis (WGCNA) was used to analyze the relationship between DEGs and clinical dimensions. According to the results of WGCNA, in total, 423 genes with high connectivity in the clinically significant module were identified as hub genes. Then, six common hub genes were obtained from the intersection of the ferroptosis-related gene, DEGs from GSE75010, and hub gene by WGCNA. Finally, we analyzed the correlation between the expression of key ferroptosis DEGs and clinicopathological characteristics, and verified by immunohistochemistry and Western blot. Our results may help to elucidate the potential role of the ferroptosis process in placental pathology and provide a novel perspective for clinical diagnosis and treatment of preeclampsia.

## Materials and methods

### Data acquisition and processing

**FIGURE 1:**
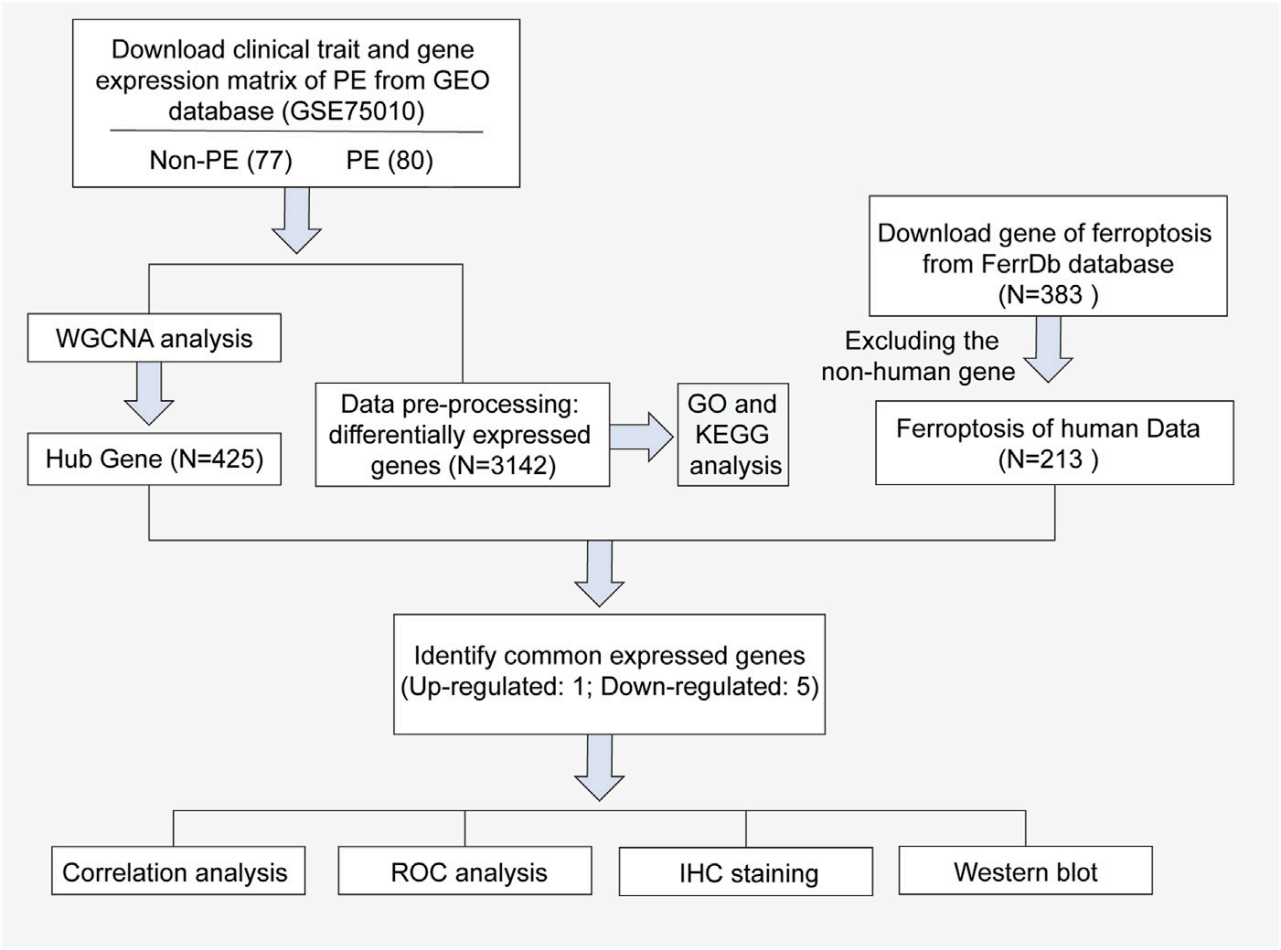
Workflow of searching hub genes in preeclampsia.

RNA sequencing dataset (GSE75010) and corresponding clinical data of preeclampsia patients and non-preeclampsia patients were downloaded from the GEO databases (https://www. ncbi. nlm. nih.gov/geo). The dataset contains 77 normal placental tissues and 80 preeclampsia placental tissues. The characteristics of the patients in the two groups, including ultrasound parameters, are presented in Table 1 **.** The corresponding ferroptosis-related gene list was downloaded from FerrDb (https://www.zhounan.org/ferrdb/legacy/index.html) (Zhou and Bao, 2020). In total, 383 ferroptosis-related genes were identified, including 150 drivers, 110 suppressors, and 123 markers. Non-Homo sapiens genes were identified and excluded from further analyses. The flowchart for data processing is shown in Figure 1.

**TABLE 1:** Clinical information and sample size for the GSE75010 dataset.

Characteristic	Non-PE (*n* = 77)	PE (*n* = 80)	*p*
Maternal age, y	33.18 ± 5.35	33.19 ± 5.89	0.134
BMI, kg/m^2^	24.49 ± 4.84	26.50 ± 5.64	0.026
Gestational week, weeks	33.99 ± 4.73	32.30 ± 3.57	0.012
Maximum systolic blood pressure, mm/Hg	136.12 ± 23.53	169.79 ± 18.11	<0.001
Maximum diastolic blood pressure, mm/Hg	85.27 ± 14.68	107.28 ± 9.73	<0.001
Mean arterial blood pressure, mm/Hg	102.22 ± 16.87	128.11 ± 11.21	<0.001
Mean uterine pulse index	1.56 ± 0.50	1.72 ± 0.51	0.317
Umbilical pulse index	1.30 ± 0.46	1.39 ± 0.38	0.301

### Differentially expressed genes

Differential expression analysis was carried out using the R*/*limma package. Significant results were determined based on an FDR-adjusted *p*-value ≤ 0.01. To obtain more differentially expressed genes (DEGs), |LogFC >0.1| and *p*-value <0.01 were set as the threshold. After that, we used the FerrDb database to download the ferroptosis-related gene list and intersected it with DEGs to obtain common ferroptosis-related DEGs between the preeclampsia patients and non-preeclampsia patients. Visualization of the DEGs including the volcano plot, heatmap, and Venn diagram was achieved using *R* software. A protein interaction network (PPI) is a method to calculate the correlation of each pair of interaction proteins by the coefficient of gene expression profiles to filter the protein interaction network. The result was downloaded from STRING, and then imported into the Cytoscape V3.9.1 software to find the key node genes by CytoHubba plug-in. Required confidence (combined score) > 0.4 was used as the minimum interaction score. According to the connectivity, nodes with degree ≥5 were identified as hub nodes.

### Functional enrichment and pathway of DEGs

Functional enrichment analysis and pathways enrichment analysis of the DEGs, including biological processes (BPs), cellular components (CCs), molecular functions (MFs), and pathway analyses, were performed using the clusterProfiler package in Bioconductor (http://bioconductor.org/packages/release/bioc/html/clusterProfer.html). Using the human genome as a background reference, *p* < 0.05 and enriched gene numbers (count) ≥ 2 were considered as cut-off values. Finally, the bubble diagram was used to show the results of enrichment analysis.

### WGCNA analysis

The expression profile dataset was used to screen genes with median absolute deviation (MAD) ≠ 0, subsequently; the co-expression network of these genes was constructed by WGCNA. First, the person correlation matrices and average linkage clustering were both performed for all pair-wise genes. Then, the Pearson correlation matrix was transformed into a weighted adjacency matrix using a power function f(x) = xβ based on a soft-thresholding parameter *β*. As a soft-thresholding parameter, *β* was able to emphasize strong correlations between genes and penalize weak correlations, thus ensuring the scale-free network. After choosing the power of six, the weighted adjacency matrix was transformed into a topological overlap matrix (TOM) with the topological overlap (TO)–based dissimilarity (1-TOM). Finally, average linkage hierarchical clustering was conducted to classify genes with similar expression patterns into modules according to the TOM-based dissimilarity with a minimum size (genome) of 30 for the gene dendrogram. To further analyze the module, a cut line was selected for the module dendrogram and a number of modules were merged using the function moduleEigengenes. In addition, the modules were merged if the distance is less than 0.25, and finally obtained 17 co-expression modules. Finally, we selected the hub gene according to the value of the gene signature (GS) and module membership (MM) (GS > 0.6, MM > 0.6, GS *p* value <0.05, and MM *p* value <0.05).

### Western blot

Placental tissues (5 mm × 5 mm × 5 mm) were obtained within 15 min after delivery, avoiding infarcted, necrotic, and calcified areas. Then, the tissues were washed with cold PBS to remove maternal and fetal blood. Placental tissues were snap frozen in liquid nitrogen for 10 min and were then stored at −80°C until use. Western blot analysis was performed following standard procedures. Placental tissues were weighed and homogenized in RIPA lysis buffer (100 mg tissue per milliliter) containing a protease inhibitor in a mini bead–based homogenizer for 2 min at 4,000 rpm. The homogenate was centrifuged at 130,000 *g* for 15 min at 4°C, and the supernatant was utilized as the total placenta homogenate. Protein concentrations were detected using the BCA protein assay kit (Thermo Fisher Scientific, Inc.). Total protein (30 µg/lane) was loaded into each well by SDS-PAGE using a 10% gel and then transferred to a polyvinylidene fluoride (PVDF) membrane. The membrane was then blocked with 5% nonfat dry milk for 1 h at room temperature. Following overnight incubation at 4°C with mouse anti-NQO1 antibodies (1:3,000, Cat. ab28947, Abcam) and rabbit anti-SRXN1 (1:1,000, Affinity Biosciences Cat# DF12028, RRID:AB_2844833). Subsequently, the membrane was incubated with the horseradish peroxidase–conjugated anti-mouse IgG and anti-rabbit IgG (1:1,000, Cell Signaling Technology, United States) secondary antibody for 2 h at room temperature and detected by chemiluminescence. β-Actin (1:3,000, cat. ab8226, Abcam) was used as a loading control.

### Immunohistochemistry

Human placental tissues were collected in accordance with the policy of the Ethics Committee of the First Affiliated Hospital of Jinan University. These tissues were fixed in 4% paraformaldehyde for 24 h following a standard protocol, then dehydrated and embedded in paraffin. Next, paraffin sections were sliced on microtome at a thickness of 5 μm. Mouse anti-NQO1 antibodies (1:200, Cat. ab28947, Abcam) and rabbit anti-SRXN1 (1:100, Affinity Biosciences Cat# DF12028, RRID:AB_2844833) were used as primary antibodies, and anti-mouse IgG or anti-rabbit IgG was used as a secondary antibody (1:1,000, Cell Signaling Technology, United States). The reaction was revealed using Novolink Polymer (Leica Mycrosystems, Newcastle upon Tyne, United Kingdom) followed by diaminobenzidine (DAB, Dako, Carpinteria, CA, United States) as a chromogen. The sections were then counterstained with Mayer’s hematoxylin. The results of the statistical analysis of immunohistochemistry were expressed by relative optical density (IOD).

### Statistics

All statistical analyses are shown as the means ± SD. *R* software (version 4.1.2) and GraphPad software were used to analyze the data. The normally distributed continuous variables are expressed as means ± standard deviations and the significance between the means was tested using the Student’s *t*-test. Pearson correlation analysis was used to analyze the relationships between clinical characteristics and gene expression. The receiver operating characteristic (ROC) curve and area under the curve (AUC) value analysis was performed to evaluate the diagnostic accuracy of gene expression levels for preeclampsia. A *p*-value <0.05 was considered statistically significant.

## Results

### Differentially expressed genes in the placenta samples

**FIGURE 2:**
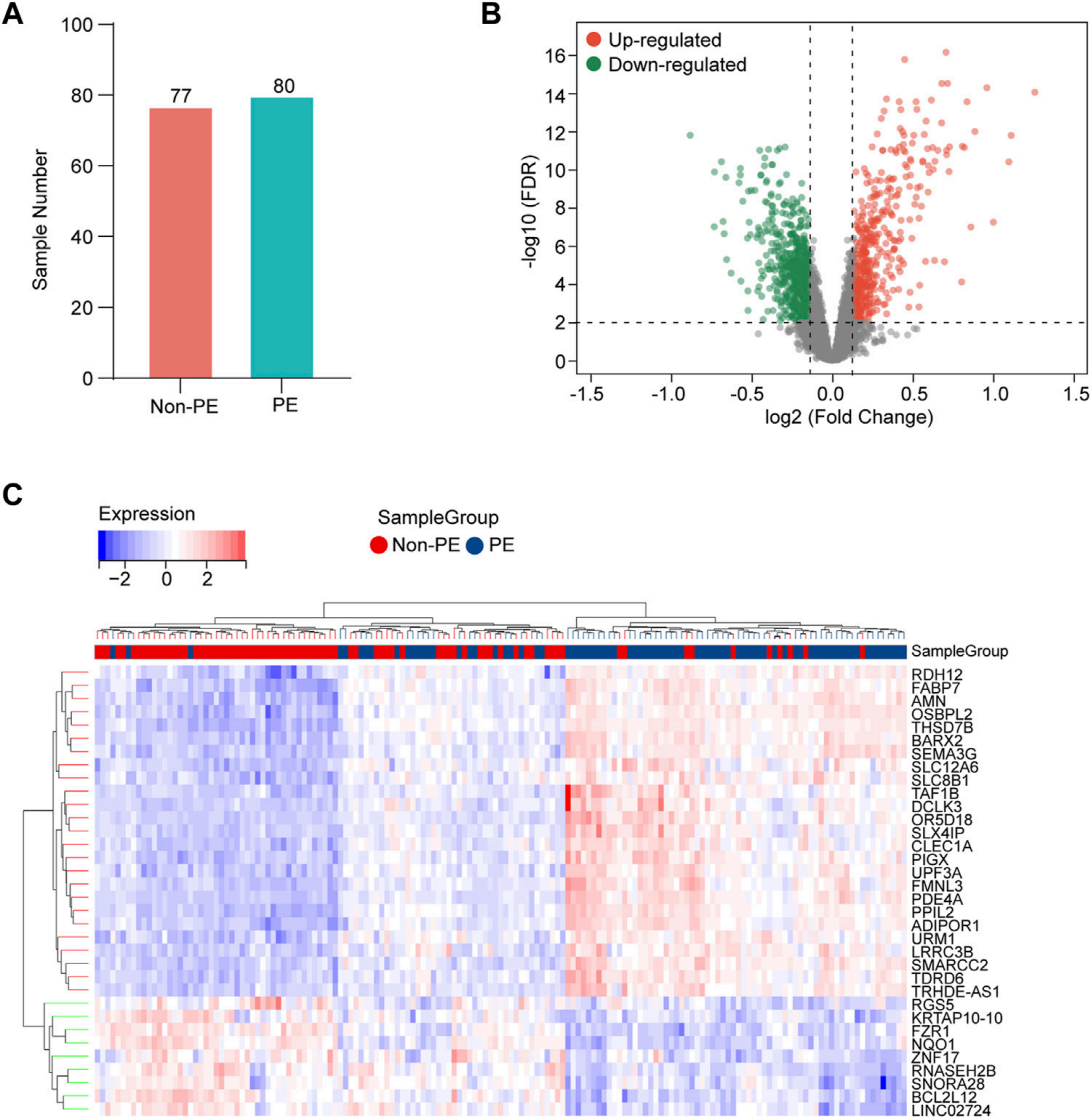
Identification of DEGs in the placenta samples. **(A)** Information of sample sizes. **(B)** Volcano plot. Red dots represent upregulated genes, gray dots represent non-significant genes, and green dots represent downregulated genes. **(C)** Heatmap for hierarchical clustering of DEGs in non-PE group and PE group.

The gene expression data in placental tissues from patients with non-PE and PE were downloaded from dataset GSE75010 in the Gene Expression Omnibus (GEO). The dataset contains a total of 157 expression data, including 77 non-PE placenta samples and 80 PE placenta samples (Figure 2A). Data normalization and differential gene expression analysis were performed using the DEGseq package in Rstudio. The results indicated that there is a total of 3,142 DEGs, including 1,468 upregulated genes and 1,674 downregulated genes. The DEGs were visualized using a volcano plot (Figure 2B). The top 15 upregulated genes and the top 15 downregulated genes are shown in Table 2. The expression of the top 34 DEGs listed by corrected *p*-values was shown by heatmap (Figure 2C).

**TABLE 2:** Top 30 DEGs in preeclampsia human placenta tissues (GSE75010).

Symbol	logFC	*p*-Value	
SMARCC2	0.707,527,378	7.29E-17	Up
SLC25A6	0.451,058,661	1.76E-16	Up
PPIL2	0.717,310,522	3.13E-15	Up
FABP7	0.681,503,942	3.13E-15	Up
PDE4A	0.961,484,928	5.23E-15	Up
OSBPL2	1.259,203,516	9.05E-15	Up
DPP3	0.338,409,602	2.05E-14	Up
TDRD6	0.616,869,246	2.33E-14	Up
SRD5A1	0.417,177,913	2.87E-14	Up
FBXO15	0.521,892,281	2.87E-14	Up
UPF3A	0.838,120,857	2.87E-14	Up
THSD7B	2.12,038,427	2.87E-14	Up
CEP126	0.526,948,466	7.18E-14	Up
FLJ20712	0.427,232,116	7.42E-14	Up
CCSER1	0.323,206,751	8.77E-14	Up
FZR1	−0.88,104,554	1.62E-12	Down
GPC1	−0.291,232,791	6.74E-12	Down
TMEM185B	−0.32,535,808	7.88E-12	Down
ZNF804A	−0.393,918,297	9.06E-12	Down
PLEKHA3	−0.339,145,447	1.01E-11	Down
GABARAPL1	−0.448,827,999	1.01E-11	Down
DISP1	−0.417,664,388	2.49E-11	Down
BCL2L12	−0.686,528,563	3.99E-11	Down
EVI5L	−0.369,314,289	5.70E-11	Down
SPDYC	−0.374,522,994	5.70E-11	Down
ZNF397	−0.419,417,572	6.45E-11	Down
LINC00687	−0.568,654,721	8.88E-11	Down
NQO1	−0.729,958,066	1.35E-10	Down
SRSF7	−0.282,884,099	1.87E-10	Down
ZNF486	−0.441,868,884	1.97E-10	Down

### GO and KEGG pathway enrichment analysis of DEGs

**FIGURE 3:**
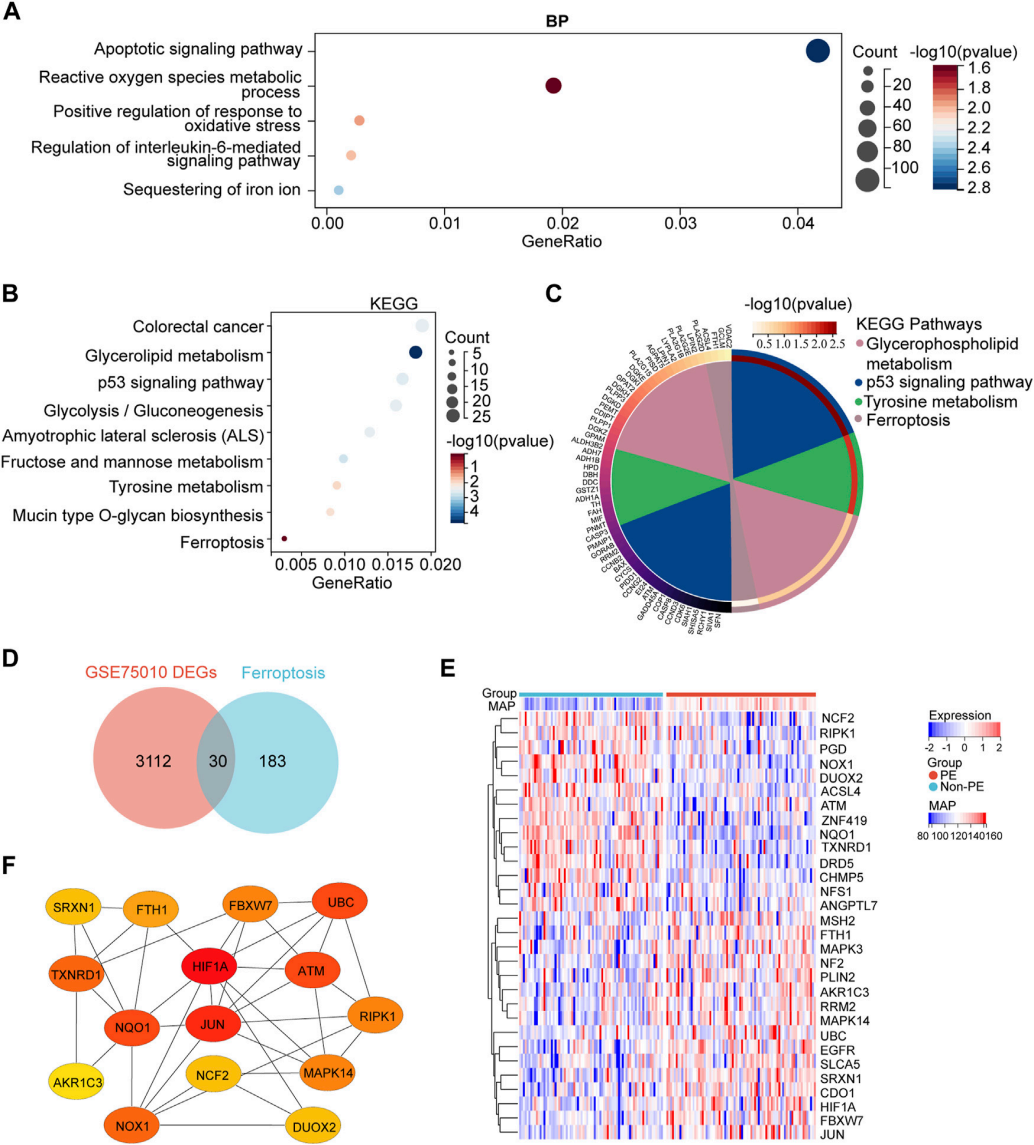
GO and KEGG enrichment analyses of DEGs and the identification of the ferroptosis-related DEGs genes. **(A)** Bubble plots of the biological process of GO analysis **(B)** Bubble plot of the KEGG pathway enrichment of DEGs. **(C)** Circle plot and network visualizing the KEGG pathway **(D)** Venn diagram of ferroptosis differentially expressed genes. Identification of ferroptosis-related DEGs by the intersection of ferroptosis dataset with GSE75010. **(E)** Heatmap depicts the expression levels of ferroptosis-related DEGs among the patients with different MAP. **(F)** Top 15 hub ferroptosis-related DEGs distinguished using color shading from yellow to red, according to the score.

To understand the biological processes in which these DEGs participated, GO enrichment analysis was conducted. The GO enrichment analysis results for the biological process showed that DEGs were mainly enriched in the apoptotic signaling pathway, reactive oxygen species metabolic process, positive regulation of response to oxidative stress, regulation of the interleukin-6–mediated signaling pathway, and sequestering of iron ion (Figure 3A). The KEGG pathway found that DEGs were mainly enriched in the colorectal cancer, glycerolipid metabolism, p53 signaling pathway, glycolysis/gluconeogenesis, amyotrophic lateral sclerosis (ALS), fructose and mannose metabolism, tyrosine metabolism, mucin-type O-glycan biosynthesis, and ferroptosis (Figures 3B,C).

Next, to identify ferroptosis DEGs, 213 ferroptosis-related genes were downloaded from the FerrDb and intersected with the DEGs from GSE75010. The Venn plot shows the intersection of DEGs and ferroptosis-related genes (Figure 3D). In total, 30 ferroptosis-related DEGs were found, including 15 upregulated and 15 downregulated genes (Table 3). The expression levels of 30 ferroptosis-related DEGs in patients with different mean arterial blood pressure (MAP) were displayed by the heatmap hierarchical clustering (Figure 3E). Finally, the ferroptosis-related DEGs were analyzed using the STRING online database, and a PPI network with 15 nodes was obtained. Cytoscape plugin cytoHubba was used to identify hub genes in the network (Figure 3F).

**TABLE 3:** Ferroptosis differentially expressed genes of preeclampsia.

Symbol	LogFC	*p*.Value	
SRXN1	0.387,457,282	3.81525E-08	Up
EGFR	0.160,307,877	2.76892E-06	Up
CDO1	0.114,219,732	3.92445E-06	Up
SLC7A5	0.206,715,178	2.71739E-05	Up
HIF1A	0.069,072,478	9.56038E-05	Up
NF2	0.099,292,518	0.000110819	Up
JUN	0.139,921,123	0.000198072	Up
MAPK14	0.071,954,526	0.00057268	Up
FBXW7	0.12,554,816	0.000679231	Up
FTH1	0.077,742,087	0.00068564	Up
PLIN2	0.054,795,716	0.001486913	Up
AKR1C3	0.069,526,121	0.00199118	Up
UBC	0.238,814,977	0.004743385	Up
MAPK3	0.052,422,193	0.006558569	Up
RRM2	0.070,809,687	0.008472648	Up
NQO1	−0.729,958,066	7.92E-13	Down
NOX1	−0.259,231,438	3.85E-10	Down
DRD5	−0.355,072,779	2.06E-09	Down
TXNRD1	−0.242,552,492	2.70E-07	Down
SETD1B	−0.161,766,143	1.33E-06	Down
ACSL4	−0.149,068,233	1.60E-06	Down
ZNF419	−0.251,278,559	2.21E-06	Down
CHMP5	−0.147,018,784	8.83E-06	Down
DUOX2	−0.271,599,394	3.56E-05	Down
RIPK1	−0.119,619,935	4.51E-05	Down
NCF2	−0.112,927,977	7.44E-05	Down
ATM	−0.086,181,541	0.003,350,464	Down
ANGPTL7	−0.061,475,419	0.004,935,783	Down
PGD	−0.052892171	0.006,959,008	Down
NFS1	−0.097,714,247	0.009,068,991	Down

### WGCNA and identification of critical modules

**FIGURE 4:**
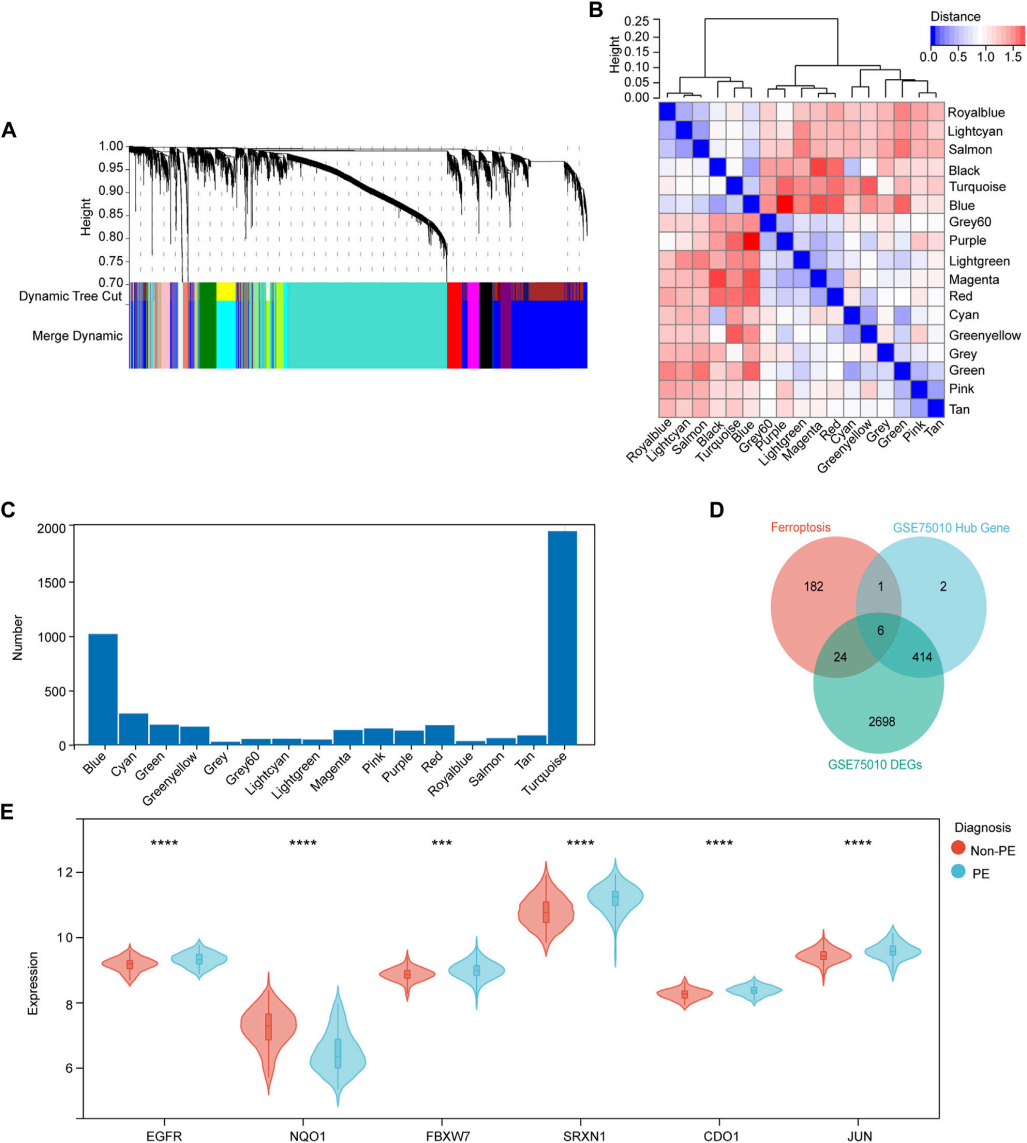
Identification of candidate ferroptosis-related hub genes in the placenta of preeclampsia. **(A)** Dendrogram of all genes clustered based on a dissimilarity measure (1- TOM). Each branch represents a gene and each color represents a co-expression module. **(B)** Module eigengene dendrogram and heatmap of eigengene adjacency. **(C)** Number of genes in each co-expression module. **(D)** Venn diagram shows common genes in the three gene sets. **(E)** Violin plot showing expression levels of six hub genes in non-PE and PE women. *p < 0.05, **p < 0.01, ***p < 0.001, ****p < 0.0001.

WGCNA was used to group the genes into modules (clusters) according to similarly expressed genes. Finally, 4,738 genes were generated for further analysis, with the identification of 17 modules (Figure 4A). The correlations among these modules were confirmed through analysis of hierarchical clustering, adjacency relationships, and heatmaps (Figure 4B). In addition, the turquoise module contained the largest number of genes (Figure 4C). In addition, Pearson correlation coefficient analysis was performed to connect each of the co-expression modules with clinical traits including diagnosis, age, and BMI. It was observed that the green module exhibited the highest negative correlation (r = −0.58; *p* = 1.0e-15) and the blue module (r = 0.62; *p* = 4.0e-18) exhibited the highest positive correlation focusing on the diagnosis of PE (Supplementary Figure S1A). This result indicates that MM and GS scores strongly positively correlated with each other in the blue and green modules (Supplementary Figures S1B,C). According to the cut-off criteria (|MM| > 0.6 and GS > 0.6), in total, 423 genes with high connectivity in the clinically significant module were identified as hub genes. Finally, six common hub genes were obtained from the intersection of ferroptosis-related gene, DEGs from GSE75010, and hub gene by WGCNA (Figure 4D). The obtained genes are EGFR, NQO1, FBXW7, SRXN1, CDO1, and JUN, respectively (Figure 4E).

### Hub gene expression and correlation with MAP and umbilical PI

**FIGURE 5:**
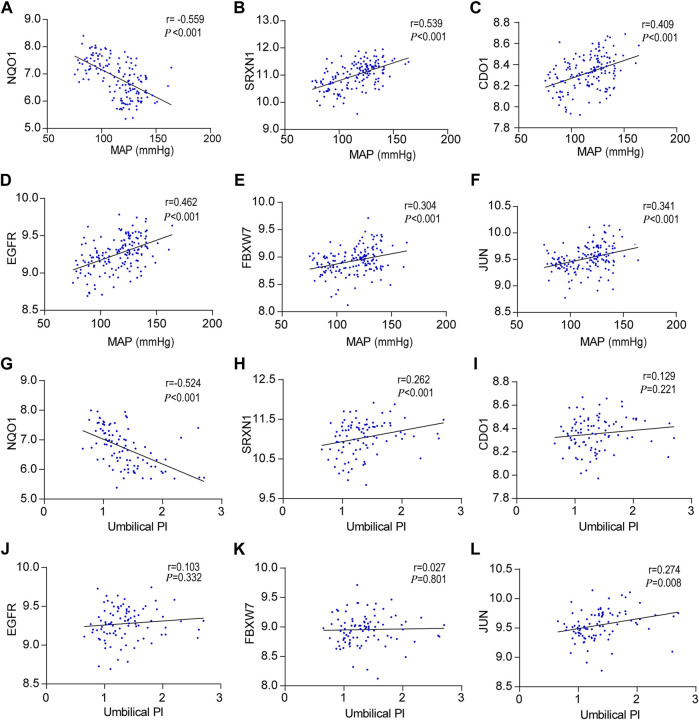
Correlation between hub genes expression levels and clinical features, **(A–F)** Pearson correlation plots between MAP and NQO1, SRXN1, CDO1, EGFR, FBXW7, and JUN. **(G–L)** Pearson correlation plots between umbilical PI and NQO1, SRXN1, CDO1, EGFR, FBXW7, and JUN.

To further investigate the relationship between hub genes and the clinical features of the patients, Pearson’s correlation was used and revealed a statistically significant correlation between MAP and NQO1 (r = −0.559, *p* < 0.001), SRXN1 (r = 0.539, *p* < 0.001), CDO1 (r = 0.409, *p* < 0.001), EGFR (r = 0.462, *p* < 0.001), FBXW7 (r = 0.304, *p* < 0.01), and JUN (r = 0.341, *p* < 0.01) (Figures 5A–F). In addition, based on the aforementioned analysis, the correlation between hub genes and umbilical pulse index (PI) in patients was analyzed by the same statistical approaches. The results revealed that umbilical PI was negatively correlated with NQO1 (r = −0.524, *p* < 0.001) and positively correlated with SRXN1 (r = 0.262, *p* < 0.001), CDO1 (r = 0.129, *p* = 0.221), EGFR (r = 0.103, *p* = 0.332), FBXW7 (r = 0.027, *p* = 0.801), and JUN (r = 0.274, *p* = 0.008) (Figures 5G–L).

### Diagnostic accuracy and experimental verification of hub genes for preeclampsia

**FIGURE 6:**
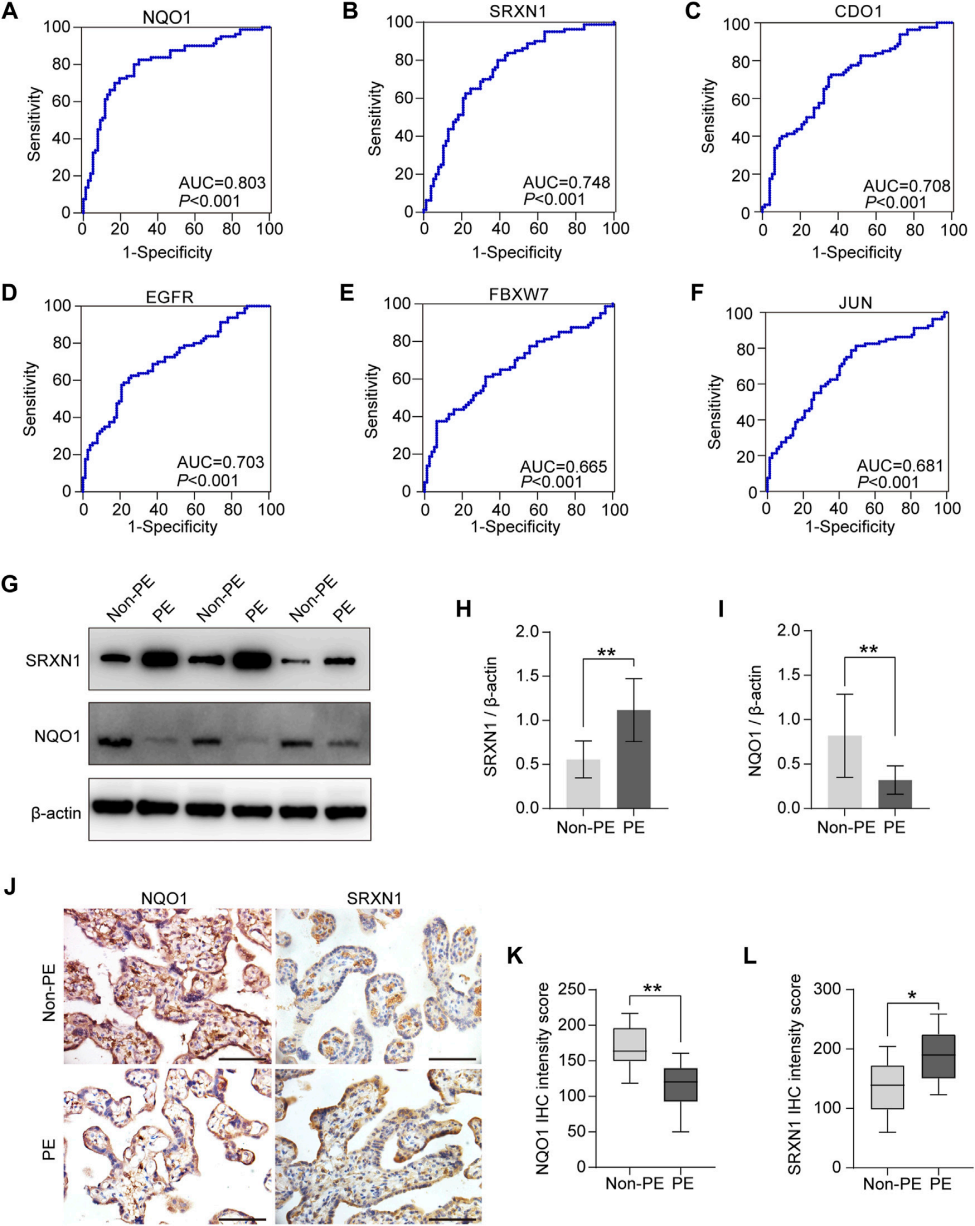
Diagnostic accuracy analysis and experimental verification of six hub genes. **(A–F)** ROC curves were plotted to examine the diagnostic potential and discriminatory accuracy of NQO1 **(A)**, SRXN1 **(B)**, CDO1 **(C)**, EGFR **(D)**, FBXW7 **(E)**, and JUN. **(F–I)** Western blot analysis of NQO1 and SRXN1 protein expression **(G)**, and gray-scale analysis of Western blot analysis results **(H–J)**. Representative images of immunohistochemical staining for NQO1 and SRXN1 in human placenta from normal pregnancy and preeclampsia. **(K,L)** Statistical analysis of immunohistochemistry results in non-PE placenta tissues (*n* = 15) and PE placenta tissues (*n* = 15). IHC means immunohistochemistry. *p < 0.05; **p < 0.01; ***p < 0.001. Scale bars = 50 μm in **(J)**.

Regarding the potential of markers for discriminating preeclampsia patients from normal pregnant women, the ROC analysis revealed that the AUC values of the NQO1, SRXN1, CDO1, EGFR, FBXW7, and JUN are 0.803, 0.748, 0.708, 0.703, 0.665, and 0.681, respectively (Figures 6A–F). The results showed that the top two genes with the highest diagnostic accuracy are NQO1 and SRXN1.

As described earlier in the previous section, the correlation analysis between the clinical characteristics of patients and six hub genes also indicated that the expression level of NQO1 and SRXN1 were associated with clinicopathological features, MAP, and umbilical PI. Therefore, NQO1 and SRXN1 were selected to be validated by Western blot and immunohistochemistry. Western blot analysis revealed that the expression levels of NQO1 in placental tissues from patients with preeclampsia were significantly lower than normal control placentas (*p* < 0.05). Meanwhile, SRXN1 expression levels were significantly increased in preeclampsia placentas compared with normal control placentas (*p* < 0.05) (Figures 6G–I). Immunohistochemistry analysis of placenta tissues showed that the NQO1 expression decreased in preeclampsia, whereas SRXN1 expression increased (Figures 6J–L).

## Discussion

In this work, a number of key genes involved in ferroptosis were identified, which aimed to provide clues for further studying the role of ferroptosis in preeclampsia. Our study obtained 3,142 DEGs, including 1,468 upregulated and 1,674 downregulated DEGs. Then, the GO enrichment analysis and KEGG pathway of DEGs were performed using the clusterProfiler package in Bioconductor, and the results indicated that these genes are involved in some pathways related to ferroptosis. These data suggest that ferroptosis may participate in the pathogenetic process of preeclampsia. In addition, 30 ferroptosis-related DEGs were obtained, including 15 upregulated and 15 downregulated genes from the intersection of DEGs and FerrDb. Next, two modules and six hub genes significantly related to the diagnosis of preeclampsia were identified through WGCNA. Finally, among these hub genes, we eventually selected NQO1 and SRXN1 for further validation based on the relationship between expression levels and clinicopathological features and diagnosis value using the ROC curve. In summary, our study may provide an interesting insight into the pathological mechanism of preeclampsia from the perspective of bioinformatics analysis.

Ferroptosis, an iron-dependent and regulated form of cell death, is characterized by the intracellular accumulation of lipid hydroperoxides (Yu et al., 2022). In the process of cell ferroptosis, free iron and iron-containing lipoxygenase enzymes are responsible for the oxidation of polyunsaturated fatty acids (PUFAs), causing the formation of lipid radicals and reactive oxygen species (ROS) (Johannsmeier et al., 2018; Li et al., 2020). One of the functions of cystine–glutamate transporter (systemXc-) is to eliminate lipid ROS formation through GPX4 and its dysfunction would cause accumulation of lipid ROS, finally causing ferroptosis (Yi et al., 2020). Therefore, inhibition of systemXc-, GSH depletion, and GPX4 inactivation become the key links in inducing ferroptosis in cells. The placenta plays a key role in fetal development, maternal–fetal communication, maternal homeostasis, and pregnancy adaptation to injuries (Liang et al., 2018; Hao et al., 2020; Guo et al., 2021). It is generally known that early pregnancy disruptions in placentation and placental function leads to insufficient spiral artery remodeling and, as a result, prolonged placental ischemia (Shao et al., 2017; Kelemu et al., 2020). Importantly, some studies have already revealed that ferroptosis may play a key role in the placental dysfunction. During the development of the placenta, their situations may induce ferroptosis, including hypoxia-reoxygenation transitions (Ducsay et al., 2018; Chakraborty et al., 2020), iron ion enrichment in placental trophoblasts (Fisher and Nemeth, 2017; Cao and O’brien, 2013; Sangkhae et al., 2021), trophoblastic lipid peroxidation (Beharier et al., 2021; Liao et al., 2022), and lower levels of ferroptosis-mitigating guards (Peng et al., 2016). In our study, ferroptosis-related processes were enriched, including reactive oxygen species metabolic process, positive regulation of response to oxidative stress, and sequestering of iron ion. Preeclampsia is proposed to be a consequence of insufficient trophoblast invasion and incomplete vascular remodeling, which lead to reduced placental perfusion and impaired placentation, and causes systemic pathophysiological changes in the maternal circulation (Li et al., 2016). Therefore, we can hypothesize that the imbalance of iron homeostasis and oxygen regulation at the maternal–fetal interface may lead to abnormal trophoblast invasiveness, resulting in insufficient spiral arterial remodeling and pathological damage in preeclampsia.

Inflammatory responses are an essential part of innate immune responses to changes in the internal and external environment (Pathipati and Ferriero, 2017). When cells were subjected to stress, the essential arachidonic acid (AA) is released from phospholipids in cells by the action of phospholipase A2 and is subsequently broken down into several biologically active substances by lipoxygenases (LOXs) and cyclooxygenases (COXs) which activate the inflammatory response (Song et al., 2018; Fredman and Macnamara, 2021). AA is converted to various biologically active prostaglandins (PGs) by COXs. Studies have revealed that ferroptosis can induce the expression of COX2 to increase the inflammatory factors release (Yang et al., 2014). Ferroptosis also triggers inflammatory responses by releasing immunogenic damage–associated molecular patterns (Sun et al., 2020). In addition, GPX4 directly inhibits LOXs and COXs by reducing the intracellular redox state activation (Sun et al., 2020). Preeclampsia may be the result of aberrant immune system activation in pregnant women, which is characterized by endothelial dysfunction and excessive inflammation (Albonici et al., 2020). In our study, we found that regulation of interleukin-6–mediated signaling pathway was enriched in preeclampsia. Some studies have found that IL-6 can induce ferroptosis of cells involved in the occurrence and development of diseases (Han et al., 2021; Li et al., 2022). However, the specific role of IL-6-regulated ferroptosis in preeclampsia has not been reported and needs to be further explored. In KEGG enrichment analysis, we found that the p53 signaling pathway was enriched in the DEGs. The p53 gene is an important tumor suppressor gene, and its mediated cell cycle inhibition, senescence, and apoptosis play an important role in the occurrence and development of tumors (Coskun et al., 2022). Some recent studies have revealed that the relationship between the ferroptosis process and the TP53 gene, implying that the P53 protein may play a role in the regulation of the ferroptosis process (Blaber et al., 2015; Jiang et al., 2015; Chen et al., 2020; De Souza et al., 2021). However, the specific molecular mechanism of p53-regulated ferroptosis in preeclampsia still needs to be further elucidated.

In the present study, six DEGs were identified as the most significant hub genes, and NQO1 and SRXN1 were selected for further validation by immunohistochemistry and Western blot. NQO1 encodes NAD(P)H dehydrogenase, which is mainly responsible for protecting cellular membranes from peroxidative injury (Zhang et al., 2018). Following hydride transfer from NAD(P)H to the FAD co-factor in NQO1, NQO1 assumes a reduced shape under typical settings where NAD(P)H levels are abundant (Siegel et al., 2021). Numerous studies have shown that NQO1 plays a crucial role in the antioxidant defense mechanisms (Onda et al., 2015; Zhao et al., 2017; Ziemba et al., 2018), however, how NQO1 regulates ferroptosis in preeclampsia still needs to be further studied. As an antioxidant, SRXN1 may be utilized as an antioxidant defense system involved in the maintenance of oxidative homeostasis (Lv et al., 2020). The expression of SRXN1 is increased after the occurrence of cells damaged by oxidative stress. Recent studies have shown that SRXN1 plays pivotal roles in the regulation of ferroptosis in some cancers (Chen et al., 2021). In our study, we confirmed that SRXN1 was elevated in human preeclampsia placentas. Together, this evidence suggests that placental tissues are susceptible to lipoperoxidation, and this will be further exacerbated if antioxidant defenses are depleted.

In the present study, we identified several hub genes that are closely associated with ferroptosis in preeclampsia and some of which until recently were not mentioned in the literature in the context of the preeclampsia. These findings may provide new insights into the regulation of ferroptosis-related genes, identifying novel candidate genes. Taken together, our findings will provide relevant evidence for the role of ferroptosis in preeclampsia. However, further studies are necessary to explore the deeper mechanisms of placental ferroptosis and its role in the preeclampsia pathogenesis.

## Data Availability

The datasets presented in this study can be found in online repositories. The names of the repository/repositories and accession number(s) can be found in the article/Supplementary Material.
