# Supplementary figures and images for: Ferroptosis-related gene expression in the pathogenesis of preeclampsia
Source: Front Genet. 2022 Aug 17;13:927869. doi: 10.3389/fgene.2022.927869 (PMC9428486; doi:10.3389/fgene.2022.927869)

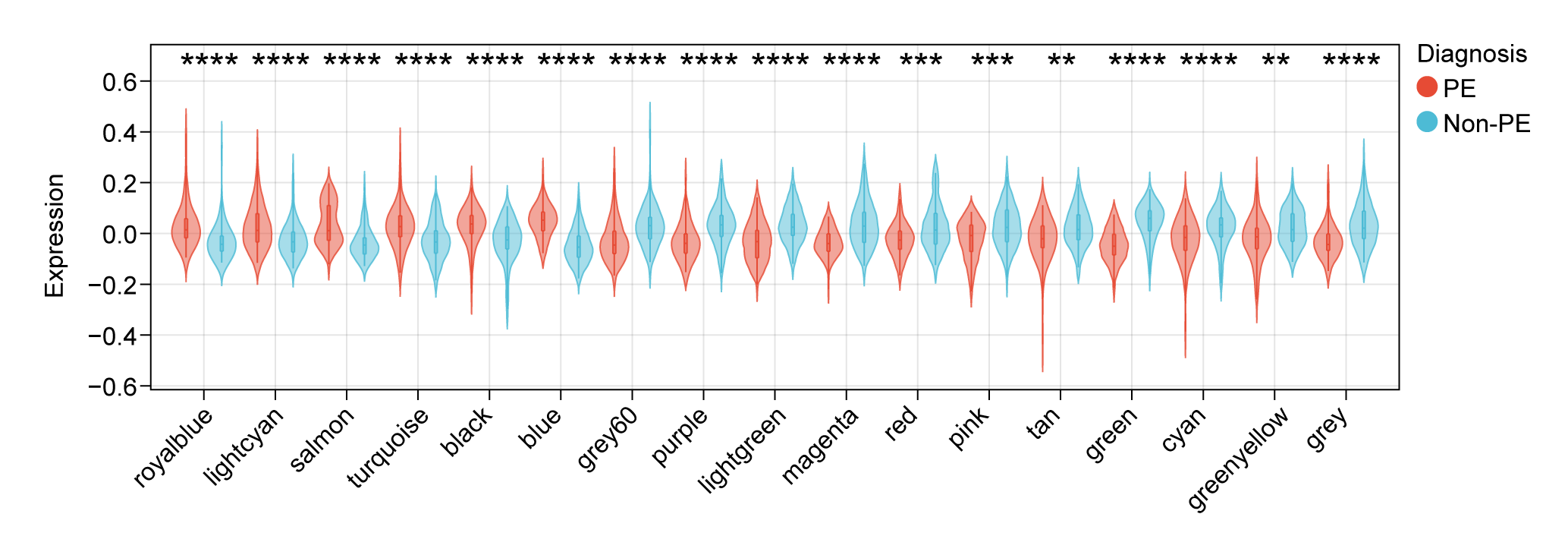

Supplement: Supplementary file 1 [file Image2.TIF]

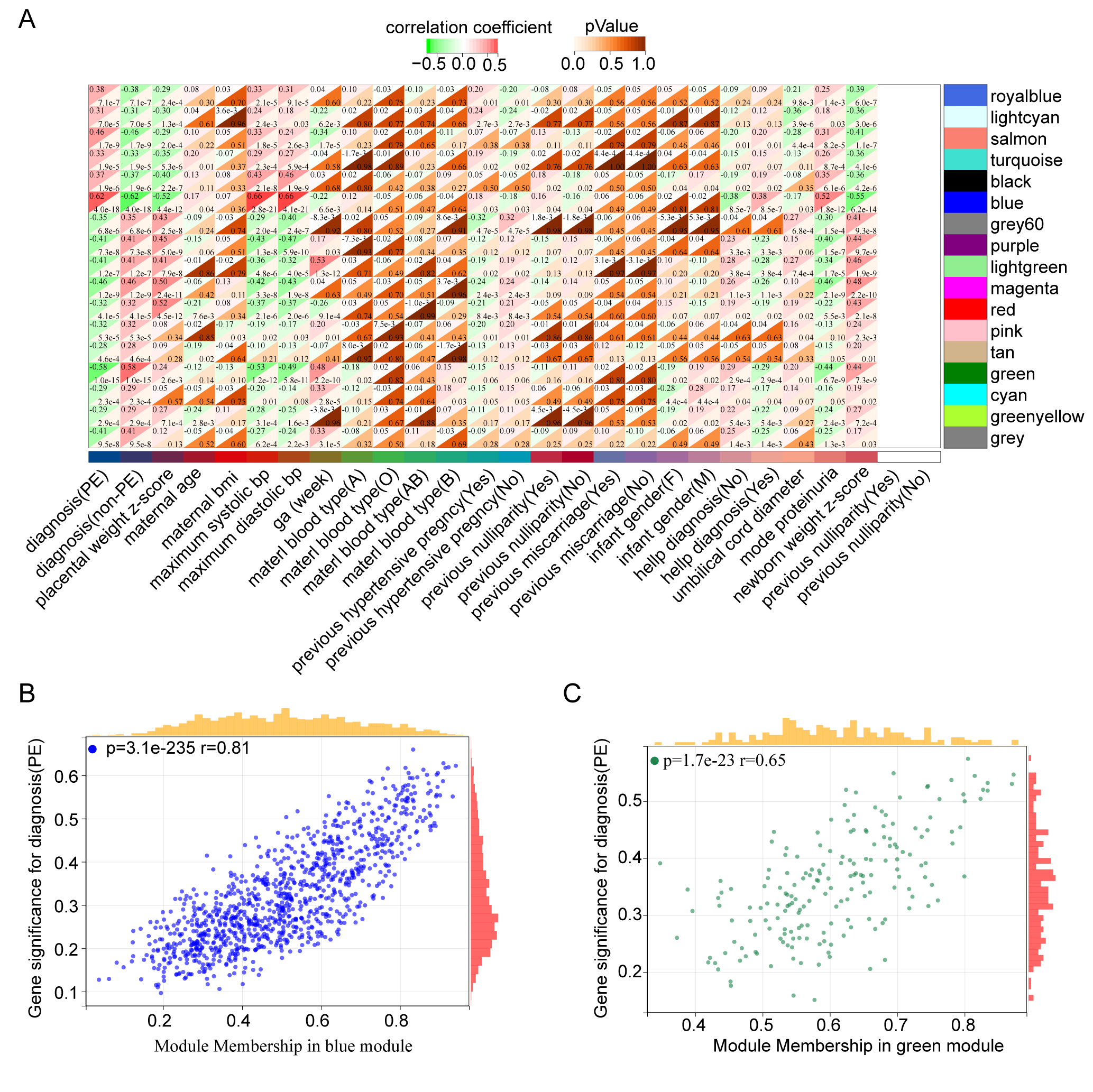

Supplement: Supplementary file 2 [file Image1.TIF]
